# Beyond paper walls: From diagnosis to action on visa inequities in global health

**DOI:** 10.1371/journal.pgph.0006964

**Published:** 2026-07-29

**Authors:** Shawna Novak, Anahi Venzor Strader, Peva Gbagornah, Susana Orrego Villegas

**Affiliations:** 1 Department of Global Health and Social Medicine, Harvard Medical School, Boston, Massachusetts, United States of America; 2 The Canada International Scientific Exchange Program (CISEPO), Toronto, Canada; 3 Boston Children’s Hospital, Boston, Massachusetts, United States of America; 4 Beth Israel Deaconess Medical Center, Boston, Massachusetts, United States of America; McGill University, MALAYSIA

## Abstract

Visa inequities in global health have moved from the margins of academic discourse to the centre of a growing reform agenda. While recent literature has established that visa regimes function as structural determinants of global health participation, a critical gap remains: the solution pathways proposed to date remain dispersed across commentaries, editorials and toolkits rather than consolidated into a coordinated operational framework that institutions, conference bodies and funders can act on at scale. This Essay consolidates and builds on that emerging consensus to propose a Global Mobility Equity Framework comprising four interdependent and scalable domains: equitable convening, inclusive participation infrastructure, institutional accountability and distributed visa support systems. Drawing on documented cases spanning the International AIDS Conference, the World Health Summit, the Consortium of Universities for Global Health, and clinical training pathways for foreign medical graduates, we demonstrate that visa barriers impose cascading professional, financial and institutional costs that are systemic in pattern rather than exceptional in nature. We further show that virtual and hybrid conferencing, while promising, does not independently resolve underlying inequities related to connectivity, time zones, language access and the networking disparities that shape career trajectories. The framework we propose is not a restatement of the structural critique as that ground has been well covered. It is a citable, implementable operational response designed to guide coordinated action across global health’s institutional landscape. Adopting this framework is a necessary condition for a more representative and effective global health system.

## Introduction

Visa inequities have moved from the margins of global health discourse to the centre of a growing reform agenda. Bain and colleagues have recently demonstrated that visa restrictions function as structural determinants of global health, not as temporary administrative inconveniences, but systemic gatekeeping mechanisms that shape who participates in research, governance and knowledge production [1,2]. This reframing is important and overdue. Building on this diagnosis, scholars have begun to set out concrete solution pathways, ranging from short-, medium- and long-term reforms to a power-and-interest analysis of the stakeholders who must act [3]. What the field still lacks is not ideas but their consolidation: a single, coordinated operational framework that institutions, conference bodies and funders can adopt, finance and be held accountable to.

This essay addresses that gap. Drawing on documented cases spanning the International AIDS Conference, the World Health Summit, the Consortium of Universities for Global Health and clinical training pathways for foreign medical graduates, we demonstrate that visa barriers impose cascading professional, financial and institutional costs that are systemic in pattern rather than exceptional in nature. The personal narratives presented here are not anecdotes; they are evidence of structural failure. We then propose a Global Mobility Equity Framework comprising four interdependent domains that together constitute a coordinated, scalable response. These four cases were chosen purposively: each represents a distinct site through which global health mobility operates, a recurring disease-specific scientific congress (the International AIDS Conference), a high-level governance and policy summit (the World Health Summit), a membership consortium that structures academic global health (the Consortium of Universities for Global Health), and the longitudinal training pathways that determine who enters and advances in the workforce (clinical training for foreign medical graduates). Together they trace the mobility ecosystem across convening, governance, education and professional advancement; they are illustrative of a systemic pattern rather than an exhaustive catalogue.

The argument we make is deliberately constructive. The diagnosis is now established in the literature [1,2,4]. What global health needs next is not another account of the problem, but a citable, implementable pathway from structural critique to institutional practice.

### The scale and pattern of exclusion

The financial and professional costs of visa denial are not marginal. Pakistani researchers alone spent £5.3 million on rejected United Kingdom visas and €3.3 million on denied Schengen visas in 2023, with rejection rates of 40% and 50% respectively [5]. A 2023 Royal Society analysis found that 22 of the 30 territories for which the United Kingdom most frequently refused visitor visas were in Africa [6]. In the United States, the denial rate for F-1 visas is approximately 54% for African students compared to 9% for European students [7]. These are not incidental statistics; they are the financial architecture of exclusion.

At conferences specifically, the pattern is well documented. Velin and colleagues’ systematic review demonstrates that global health convenings remain dominated by participants from high-income countries, with visa barriers among the primary contributing factors [8]. At the 2024 CUGH Annual Conference, the Working Group on Equitable Opportunities in Clinical Education dedicated a satellite session to structural barriers including US visa regulations and conference equity [9]. At the 2018 Women Leaders in Global Health Conference hosted by the London School of Hygiene and Tropical Medicine, at least 17 researchers;14 from sub-Saharan Africa and 3 from Asia, were denied visas [10]. The International AIDS Conference in Montreal compelled organisers to reconsider conference locations entirely following widespread visa denials to delegates from the Global South [11,12]. During the World Health Summit in Berlin, senior public health leaders from Africa reported mistreatment at borders and visa barriers that prevented their participation [13]. Bandara, Deng and Pai argued in 2025 that the Global North has become structurally unsafe as a venue for global health meetings [4].

These barriers extend into training pathways. Foreign medical graduates and medical students seeking clinical training in high-income countries face substantial visa restrictions. In the United States, hands-on clinical participation is generally available only through tightly regulated, institutionally sponsored pathways, such as accredited residency or fellowship training. Many short-term electives, clerkships and observerships are limited, costly or restricted. These constraints limit professional development and access to clinical networks [14]. In contrast, trainees from high-income countries frequently access clinical experiences in low- and middle-income settings with fewer comparable barriers [3]. This asymmetry is not incidental. It reflects who is permitted to acquire professional legitimacy within the global health system.

Global mobility barriers are also increasingly commercialised: VFS Global, which operates visa-application processing in 153 countries on behalf of 68 governments, has been shown to profit substantially by charging applicants, disproportionately citizens of low- and middle-income countries, for add-on services that are difficult to refuse, with such value-added services accounting for roughly 30% of its revenue and as much as 47% in some settings [15].

### Personal narratives: Lives interrupted by bureaucracy

Beyond aggregate data, visa inequities are experienced as deeply personal disruptions with cascading professional and societal consequences. The narratives that follow are illustrative of systemic patterns, not exceptional cases. They illuminate how structural barriers operate in practice and why the institutional complacency that allows them to persist is itself a form of harm.

Dr. Adeola B., a promising epidemiologist from Nigeria, was invited to present her research on HIV/AIDS at a prestigious international conference. Despite fulfilling all formal requirements, her visa application was denied on unspecified “security grounds.” The absence of institutional support left her to navigate a complex and opaque system alone. This is a recurring pattern in which responsibility for overcoming structural barriers is placed on individuals rather than addressed collectively. Despite her continued commitment to research and advocacy, she carries what can be understood as a “double burden”: advancing scientific work while simultaneously navigating structural inequities that others are not required to confront.

Similarly, Stanley A., a climate and sustainable development specialist from Liberia, was awarded a prestigious scholarship to attend an Ivy League university. Despite his academic merit and institutional support, his visa application was denied based on concerns regarding his intent to return. This justification, commonly applied to applicants from low-income settings, illustrates how structural biases are embedded within adjudication processes. Despite intervention efforts involving academic institutions, diplomatic channels and legal advisors, the outcome remained unchanged. From an ethnographic perspective, Stanley’s experience reflects how mobility regimes are shaped not only by policy, but by broader narratives about risk, belonging and economic value that operate independently of individual merit.

The financial consequences are also significant. Applicants frequently incur non-refundable application fees, travel bookings and accommodation deposits that are unrecoverable in the event of rejection. These costs disproportionately affect individuals from resource-limited settings, further entrenching inequities in access. At a systemic level, they contribute to widening disparities in global knowledge exchange, as those most affected are also those most excluded from participation.

### Why virtual conferencing does not resolve this

A common institutional response to visa barriers is to offer virtual or hybrid participation. While digital platforms reduce some barriers and can meaningfully expand access, evidence consistently shows that they do not resolve the underlying equity problem [16].

Connectivity disparities remain substantial in many LMIC settings, and poor inclusion of online audiences in hybrid events is widely documented [17]. Time zone inequities mean that asynchronous access is not equivalent to synchronous participation, particularly for the networking and relationship-building that shape career trajectories and research collaborations [18]. Without intentional design, that incorporates low-bandwidth functionality, multilingual access, offline materials and structured networking sessions, virtual formats replicate many of the biases and exclusions inherent to in-person events [16]. Virtual access, offered as a substitute rather than a complement to in-person participation, risks becoming a form of symbolic inclusion that legitimises continued exclusion. A further, structural limitation is too often overlooked: the platforms on which virtual and hybrid convenings depend, such as Zoom, Microsoft Teams and their equivalents, are predominantly developed and owned by Global North corporations [19]. They carry their own constraints, including inaccessibility in sanctioned countries, data-sovereignty concerns over where participant data is stored and governed, and interface and language defaults that reflect high-income technical norms. A participation infrastructure that is itself owned and governed in the Global North can, if left unexamined, reproduce the very dependencies and exclusions that equitable participation is meant to dismantle [19].

This is not an argument against virtual conferencing. It is an argument that virtual participation must be designed for equity rather than offered as a response to it. This distinction is central to the framework we propose. What does meaningful virtual participation actually require? Defining this, alongside developing trackable metrics for influence, networking, and equity in hybrid global health spaces, is an urgent research and policy agenda. This framework is intended to open that conversation, not close it.

### From principles to practice: A global mobility equity framework

The recognition of visa inequities as structural barriers is now established in the literature [1,2,4]. Concrete solutions have also been proposed, from Bandara and colleagues’ short-, medium- and long-term reforms and their stakeholder power-interest analysis [3], to practical siting tools such as the Campaigns in Global Health toolkit for equitably accessible meetings [20] and crowd-sourced guidance on visa-friendly venues [21]. What is missing is a structure that consolidates these dispersed proposals into a coordinated, mutually reinforcing whole. To address this, we synthesised these existing recommendations together with the documented cases above and grouped them by the level of the system at which they act: where global health is convened, how participation is designed, who bears institutional responsibility and how support is delivered at scale. We propose a Global Mobility Equity Framework comprising four interdependent domains. These are not sequential steps; they are mutually reinforcing components of a system-level response.

### Equitable convening: Decentralising where global health happens

The geographic concentration of global health conferences in high-income countries with restrictive visa regimes is itself a structural problem. Relocating or rotating conferences to visa-accessible settings, expanding regional and South–South forums, and adopting distributed multi-hub conference models can reduce reliance on restrictive immigration systems while expanding participation [22]. This is not merely a logistical adjustment. Conferences are not neutral spaces; they are sites where agendas are set, priorities are legitimised and leadership pathways emerge [23]. Where they are held determines who can influence them. Practical instruments to guide this shift already exist and can be adopted directly, including structured methodologies for selecting visa-accessible meeting locations, such as the Campaigns in Global Health toolkit for equitably accessible meetings [20], and crowd-sourced inventories of visa-friendly venues across Africa, Asia, Latin America and the Middle East [21].

The International AIDS Society has already committed to a global rotation of its conferences as a direct response to visa-related exclusions [24]. This model should become the norm rather than the exception across global health convenings.

### Inclusive participation infrastructure: Designing equity into engagement

Geographic redistribution is necessary but not sufficient. Equitable participation also requires the intentional design of participation systems. An equity-oriented approach requires designed hybrid systems that integrate asynchronous participation, repeated scheduling across time zones, multilingual access, low-bandwidth functionality and structured networking opportunities for remote participants [17,18,25].

The evidence from pandemic-era conferencing is instructive. Conferences that saw genuine gains in LMIC participation during this period shared common features: lower registration fees, flexible scheduling, and technical support for connectivity. Those that simply moved online without redesigning participation structures replicated existing hierarchies in digital form. Infrastructure investment that supports local access hubs, connectivity support and partnerships with technology providers, is essential to making this domain operational.

### Institutional accountability: Shifting responsibility from individuals to systems

The current default places responsibility for navigating visa barriers on the individuals most harmed by them. As Pai and others have argued, this transfers the burden of structural inequity onto those least equipped to carry it [26]. Addressing this requires a shift toward institutional accountability, whereby universities, conference organisations and funders take formal responsibility for mitigating barriers to participation.

This includes financial support for visa application costs, logistical assistance with documentation, and institutional advocacy in cases of unjust denial. It also includes advocacy for visa policy reform at government and intergovernmental levels. Bandara and colleagues have outlined concrete options including dedicated visa support teams, proactive government engagement and budgeted support for Global South participants [3]. These are not aspirational recommendations; they are implementable commitments that institutions can make and be held accountable for. Bandara and colleagues frame these commitments across three horizons: immediate steps such as dedicated visa-support teams, Global South travel funds and lower registration fees; medium-term measures such as making visa equity an explicit institutional advocacy priority and creating special admission and funding tracks for scholars at risk; and long-term, governance-level reform engaging foreign ministries, multilateral bodies and high-level forums including the World Health Assembly, the World Health Summit and the Consortium of Universities for Global Health [3]. Our framework consolidates these horizons into a standing institutional function rather than an ad hoc response to each crisis.

### Distributed support systems: Operationalising equity at scale

Moving from principle to practice at scale requires infrastructure. We propose a distributed hub model in which academic institutions, non-governmental organisations and regional partners collaborate to provide decentralised visa support. These hubs would offer guidance on application processes, facilitate communication with embassies, maintain shared repositories of best practices, and collect systematic data on visa experiences to inform ongoing refinement and advocacy.

Such a model addresses the practical limitations of centralised support while strengthening regional capacity and enabling context-specific solutions. Critically, it reframes visa support as a collective institutional responsibility rather than an individual burden. This is a shift that must be reflected in organisational budgets, staffing and governance structures if it is to move beyond rhetoric.

### A call for coordinated action

Visa inequities are structural features of the global health system, not temporary disruptions. As Bain and colleagues have established, they function as durable gatekeeping mechanisms that shape who participates, whose knowledge circulates and whose leadership is cultivated [1]. This Essay has argued that the field now needs to move beyond diagnosis toward coordinated operational response.

The Global Mobility Equity Framework we propose is designed for that purpose. Its four domains: equitable convening, inclusive participation infrastructure, institutional accountability and distributed support systems, are not independent reforms. They are interdependent components of a structural redesign. Implementing any one domain without the others risks reproducing existing inequities in new forms.

The moment for this framework is now. The global health architecture is under active reform pressure, with major funders, regional bodies and academic institutions reconsidering how the system should be structured [1,4]. Mobility equity must be central to that conversation. The cost of inaction is not merely ethical; it is systemic. A global health system that cannot ensure equitable participation in its own convenings and knowledge systems is only a partial system.

## Positionality statement

This essay calls for equitable representation in global health knowledge production, and our author team should be situated accordingly. Three of the four authors are themselves from low- and middle-income countries, in Latin America (Colombia and Mexico) and sub-Saharan Africa, and carry lived and professional experience of the mobility barriers analysed here, such as visa applications, denials, and naturalization processes. All four are currently affiliated with, or completed graduate training at, a high-income-country institution. The corresponding author (S.N.) is based in Canada, does not face the same restrictions, and contributes working alongside colleagues most affected. We recognise that authors writing from within a high-income institution may not fully represent the perspectives of colleagues who are at present navigating these barriers from inside LMIC settings, and that our institutional vantage point confers access and protection that both shape and limit our account. We have sought to centre documented LMIC-led analysis and proposals throughout (for example references 1, 2, 11 and 13), and we regard broader LMIC-based co-leadership as a necessary next step that this framework is intended to enable rather than replace.
